# Enhancing Farm Dams Increases Tadpole Abundance

**DOI:** 10.1002/ece3.70803

**Published:** 2025-01-19

**Authors:** Michelle Littlefair, Ben C. Scheele, David Lindenmayer, Maldwyn J. Evans

**Affiliations:** ^1^ Sustainable Farms, Fenner School of Environment & Society The Australian National University Acton Australian Capital Territory Australia

**Keywords:** agriculture, amphibians, intervention, landscape, management

## Abstract

Understanding how agricultural and land management practices affect amphibian biodiversity is essential for conservation efforts in farmland. We investigated the impact of farm dam enhancement on tadpole abundance and growth in a highly modified farming landscape in south‐eastern Australia. We completed detailed surveys on 52 farm dams (artificial ponds or agricultural reservoirs). These dams were categorized into two groups: *enhanced* (*n* = 28), which had undergone management activities such as fencing to prevent livestock access and facilitate revegetation, and *control* (*n* = 24), which had not received any intervention and were subject to standard management practices similar to adjacent paddocks. Our findings revealed a notable increase in tadpole abundance across all species in enhanced dams, with 92% of all observed tadpoles recorded in these dams. Factors such as higher dissolved oxygen and greater riparian vegetation cover were positively associated with tadpole abundance, while high pH levels showed a negative association. We found no evidence that tadpole growth was influenced by dam enhancement. Concerningly, when the invasive fish *Gambusia holbrooki* was present, tadpoles were smaller and at earlier developmental stages. Our findings highlight the potential benefits of strategic farm dam management for improving tadpole presence in agricultural landscapes.

## Graphical abstract

The study investigated the impact of farm dam enhancement on tadpole abundance and growth in a modified farming landscape. Enhanced dams, which underwent management activities such as fencing and revegetation, exhibited significantly increased tadpole abundance compared to control dams. Factors such as dissolved oxygen and riparian vegetation positively influenced tadpole abundance, highlighting the potential benefits of strategic farm dam management for enhancing tadpole presence in agricultural landscapes.

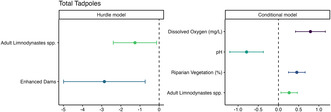

## 1 Introduction

Over recent decades, global frog populations have experienced notable declines, raising substantial ecological concerns (Collins 2010; Collins and Crump 2009; Luedtke et al. 2023). While the exact extent and causes of these declines vary across regions and species, common drivers include habitat destruction (Gallant et al. 2007), pollution (Relyea, Schoeppner, and Hoverman 2005), climate change (Mac Nally et al. 2017), disease (Olson et al. 2013; Berger et al. 2016; Scheele, Hunter, et al. 2019; Scheele, Pasmans, et al. 2019) and invasive species (Amburgey et al. 2014). The degradation and fragmentation of frog habitat restricts their ability to breed and find food, while competition and climatic change impacts behavior and survival rates. These declines have significant ecological ramifications, disrupting predator–prey relationships, nutrient cycles, and overall biodiversity within ecosystems (Zipkin et al. 2020).

Agricultural activity has transformed many natural habitats, posing severe threats to amphibian populations worldwide (Luedtke et al. 2023; Moreira, de Castilhos, and Castroviejo‐Fisher 2020). Locally, agricultural practices such as grazing and cropping directly influence the quality of farm dams, through increased nutrient runoff, sedimentation, and loss of native vegetation. For tadpoles, these conditions can lead to fluctuating water quality, a lack of shelter from predators, and limited food resources, all of which can impede their survival and growth (Mann, Hyne, Choung, and Wilson 2009). At a landscape scale, factors such as habitat fragmentation and the removal of woody vegetation further exacerbate these challenges. Farm dams often exist in isolation from other waterbodies, reducing habitat connectivity and limiting opportunities for dispersal and gene flow (Callow and Smettem 2009). Additionally, land‐use intensification, such as the conversion of pastures to cropland, introduces further pressures on freshwater habitats, including increased exposure to agricultural chemicals and reduced riparian buffer zones (Cushman, 2006; Schmidt et al. 2019). Collectively, these factors make it challenging for amphibians to complete their life cycles in highly modified agricultural landscapes.

Maintaining suitable artificial waterbodies is crucial for promoting frog conservation in farmland, especially considering the vast number of farm dams (also known as ponds or agricultural reservoirs) across agricultural regions worldwide (Malerba et al. 2023; Mantel and Hughes 2023). Enhanced farm dams that have been managed to improve their ecological health may mitigate some of the pressures of land use intensification (Lindenmayer et al. 2022). Dam enhancement can include partial or complete fencing to restrict livestock access, tree planting, and promoting the growth of native vegetation along dam edges and surrounds (Westgate et al. 2022). Fencing around a dam's perimeter reduces livestock grazing and trampling, which can affect vegetation cover (Evans et al. 2020; Lindenmayer et al. 2018) and increase sedimentation and nutrient inputs (Itebo et al., 2021). Increased vegetation provides shade, shelter, and food resources, leading to positive improvements in biodiversity, including for macroinvertebrates and frogs (Westgate et al. 2022; Littlefair et al. 2024).

Tadpoles may serve as indicators of habitat quality, relying on specific environmental conditions for survival. However, the inherent variability of waterbodies in agricultural landscapes, including fluctuations in water quality and habitat disturbance, poses substantial challenges to tadpole survival and successful amphibian recruitment (Mann, Hyne, Choung & Wilson, 2009; With et al. 2024). Collecting both tadpole and adult data is crucial for a comprehensive understanding of habitat suitability as each provides complementary information. Despite this, research in agricultural landscapes focuses predominantly on calling adult male frogs (Hazell et al. 2001; da Silva, Gibbs, and Rossa‐Feres 2011), potentially overlooking reproductive output and broader population dynamics.

In this study, we investigated relationships between farm dam structure and management practices and the reproductive success of farmland frog populations in the South West Slopes (SWS) of New South Wales, Australia. Specifically, we examined the impact of enhancing farm dams on tadpole populations. We measured water quality and vegetation cover and compared our findings with results obtained for adult frogs at the same sites (Littlefair et al. 2024) to provide a comprehensive understanding of reproductive dynamics in the region. We asked:

1. *Are there differences in tadpole abundance and growth between farm dams that have been enhanced and those that have not?*

We hypothesized that in enhanced dams tadpoles would be more abundant and be characterized by greater growth compared to tadpoles in control dams. This would reflect improved habitat quality, including reduced grazing pressure and greater vegetation cover.

We were also interested in exploring the association between enhanced dam characteristics and differences in amphibian breeding metrics such as tadpole size and developmental stage. To address this, we asked:

2 *What factors contribute to differences in tadpole abundance and growth within farm dams?*

We considered various characteristics of farm dams, including water quality, vegetation cover, and predator presence along with attributes of the surrounding landscape, including habitat connectivity, topographic wetness index, and woody vegetation cover. We anticipated that enhanced dams, due to superior habitat quality, would not only have more vegetation cover but also support a higher number of tadpoles that are larger and at a more advanced developmental stage. By addressing these two questions, we aimed to understand how farm dam management practices and landscape context of farm dams influence their habitat value for frog breeding.

## 2 Methods

### 2.1 Study Area

We surveyed 52 farm dams on 18 farms in the South West Slopes (SWS) region of New South Wales, Australia. The SWS is comprised largely of foothills and isolated ranges along the lower inland slopes of the Great Dividing Range (Benson, 2008). The SWS bioregion is one of the most intensively farmed and cleared areas in Australia, with over 96% of the area designated as freehold land and more than 80% of native woody vegetation cover cleared (State of New South Wales, 2003). The primary land use is the grazing of livestock for beef, lamb and wool, and dryland cropping of cereals and oilseed. The SWS region supports 33 of the 71 frog species found in New South Wales (Department of Climate Change, Energy, the Environment and Water, 2024), with eight species in the SWS region listed as threatened.

### 2.2 Experimental Design

We defined farm dams as relatively small (~10^2^–10^5^ m^2^) artificial (i.e., human‐made or altered) waterbodies located in agricultural settings, usually constructed for long‐term storage of water for agricultural needs. Dams varied in size, and to eliminate small dams prone to frequent drying, we used a minimum cut‐off of approximately one megaliter, with size measurements determined through a combination of satellite imagery, ground‐truthing, and landholder information. We classified dams as either “enhanced” or “control” dams. Enhanced dams (*N* = 28), which received management within the last 3–20 years, underwent either full livestock exclusion through fencing, or partial fencing, permitting restricted livestock access via a single hardened access point. At enhanced dams, native shrubs and trees were planted in the area between the fence and the dam. Control dams (*N* = 24) were subject to no management intervention and followed the same management practices as neighboring paddocks, which included either dryland cropping, grazing of cattle or sheep, or both.

Each of the 18 farms in our study included “enhanced” and “control” dams. When choosing study sites, we aimed to pair enhanced dams with a control dam of similar size, shape, and position in the landscape. This pairing design allowed us to consider similarities in climate, geometry, space, and farm management style between enhanced and control dams on the same farm.

### 2.3 Tadpole Surveys

We conducted two surveys of tadpoles during the spring (September–November) of 2021, coinciding with the peak breeding season. At each dam, the second survey was approximately 30 days after the first where possible (some dams were temporarily inaccessible during and after heavy rain). This approach allowed us to observe differences in apparent growth rates. We prioritized dams on the same or nearby farms for same‐day sampling to streamline our survey process. Among nearby dams, we randomized the specific sampling order.

We undertook tadpole surveys between 11 a.m. and 4 p.m., aligning with the warmest part of the day. We avoided conducting surveys in heavy rain, high winds, or when air temperatures were below 10°C to minimize between‐day variation in activity. We used a large mesh hand net to systematically survey designated areas within the littoral zone surrounding the dams. We sampled the cardinal points of each dam to ensure representative sampling across different habitat types. Catching tadpoles involved swiftly moving a net through the water in a figure‐of‐eight pattern, while ensuring minimal disruption to vegetation and sediment. We promptly transferred captured tadpoles to a tray of dam water for further processing. Adhering to standard hygiene protocols (NSW National Parks and Wildlife Service 2000), we counted individual tadpoles, identified them to genus level, and measured their length from snout to tail. Throughout the paper, tadpoles belonging to the *Crinia* and *Limnodynastes* genera are referred to as “*Crinia spp*.” and “*Limnodynastes spp*.” respectively. This decision was made due to difficulties in visually differentiating between closely related species such as *C. parinsignifera* and *C. signifera*, and *L. tasmaniensis* and *L. fletcherii*. We recorded developmental stages following the Gosner classification (Gosner 1960). We employed the term ‘tadpole growth’ to denote both tadpole length and Gosner stage collectively, utilizing it when not specifically addressing either measure individually. Additionally, we recorded any instances of *Gambusia holbrooki*, an invasive species known to negatively impact Australian frogs (NSW Scientific Committee, 2021), inadvertently caught during the sampling process. We completed 12 dipnets per dam.

### 2.4 Frog Surveys

In addition to tadpole surveys, we utilized data on adult frog calling collected from the same sites in the springs of 2020 and 2021 (Littlefair et al. 2024). Littlefair et al. (2024) analyzed these data to identify factors predicting adult presence, whereas our study employs this information to determine its correlation with tadpole abundance.

We obtained these data using timed visual and aural surveys conducted between 30 min after sunset to 2 am following standardized survey techniques (Crump and Scott, 1994). We did not conduct surveys during periods of heavy rain, high winds, or air temperatures below 10°C to ensure optimal vocalization and minimize between‐night variation in frog activity. Our methodology involved an initial 10‐min survey from the crest of the dam wall to record adult calls, followed by standardized visual encounter survey techniques (Crump and Scott 1994). During these surveys, we used hand‐held spotlights to search the dam's edge and surrounding habitat for a duration of 20 min, meticulously scanning beneath vegetation, fallen timber, branches, and emergent logs. We counted all encountered individuals and identified them to species level. We handled frogs only when necessary for species confirmation, following standard hygiene protocols (NSW National Parks and Wildlife Service 2000). By incorporating adult data into our analysis, we aimed to assess amphibian population dynamics across different life stages and provide a more holistic understanding of amphibian ecology in agricultural environments.

### 2.5 Dam Variables

We measured several variables that we expected to influence amphibian population metrics at each dam. These included two measures of vegetation cover, three measures of water quality, and the presence of *G. holbrooki* (Appendix S1). We gathered habitat and water quality data following the methodology outlined in Westgate et al. (2022). To assess vegetation percentage cover within the aquatic and riparian zones of each dam, we walked the dam's perimeter and visually estimated the presence of vegetation. Aquatic vegetation encompassed all of the visible submerged, floating, and emergent vegetation growing within the water, including aquatic vegetation reaching above the surface of the water. We considered riparian vegetation to be vegetation occurring between the water's edge at the survey time and the high‐water mark (Appendix S1).

We collected water samples from three randomly selected locations approximately two meters from the water's edge. At the dam level, we then combined these samples for subsequent analysis. We delivered samples to a regional water analysis laboratory accredited by the National Associate of Testing Authorities (NATA) for same‐day processing. We tested samples for metrics of water quality using standard analytical methods approved by NATA: total dissolved solids, pH, dissolved oxygen, and turbidity (Westgate et al. 2022).

### 2.6 Landscape Variables

We hypothesized that several landscape‐scale variables would influence tadpole occurrence and abundance (Appendix S1). To represent the connectivity and availability of aquatic and terrestrial habitats across the study area, we incorporated distance to the nearest waterbody and percent woody vegetation cover within a 500 m radius as landscape predictors. We also calculated a topographic wetness index (TWI), which measures hypothetical water run on based on the topography of the surrounding 500 m around each dam, excluding the water surface of the dam. We calculated the TWI using the formula ln(*α*/tan *β*), where *α* is the size of Upslope Contributing Area (UCA) per unit length of contour (i.e., pixel resolution of 10 m) and β is the slope gradient of the cell (Kopecký, Macek, and Wild 2021).

### 2.7 Data Analyses

To evaluate relationships between our predictor and response variables (total tadpole abundance, genus‐level abundance, tadpole Gosner stage, and tadpole size), we employed generalized linear mixed models (GLMMs) implemented in the “glmmTMB” package in R (Brooks, Kristensen, and van Benthem 2017). We conducted a model selection procedure using Akaike's Information Criterion corrected for small sample sizes (AICc) (Burnham and Anderson 1998), ensuring the selection of the most parsimonious models—those that provide the simplest explanation of the data while retaining predictive accuracy. For each of our five response variables, we constructed five sets of candidate models (consisting of the different predictor variables in each of the categories: dam type, water quality, dam vegetation, landscape characteristics, fauna (see Appendix S1) for descriptions of predictor variables) and then conducted independent model selection within each set of models (Appendix S2). We constructed candidate model sets using the “dredge” function in the “MuMIn” package (Bartoń 2021). We compared all subsets of models containing all predictors within each category (Appendix S3), selecting the most parsimonious models as the simplest models within ΔAICc < 2 of the lowest AICc score (Burnham and Anderson 1998). Subsequently, we integrated the predictor variables from each best‐fit model in each model set into one final model set. In this model set, we again conducted model selection on the combination of variables. To address temporal, spatial, and farm management style correlations, we included “dam” as a random intercept effect in all models. We implemented a stepwise model selection approach to avoid over‐parameterizing candidate model sets (Appendix S2).

For our count data response variables (total tadpole abundance, genus‐level abundance), we assumed Poisson error distributions. Our models exhibited zero inflation; therefore, we employed hurdle GLMMs. In a hurdle model, the first component assesses the likelihood of a zero observation (1 for observed, 0 for not observed) as a binary outcome. It is important to note that in this context, a negative outcome in the first part of the model indicates a positive effect, as it represents the likelihood of encountering zero individuals of a species. The second part of the hurdle model examined the conditional count of total individuals and of each genus observed, assuming a negative binomial error distribution. For the two growth variables (tadpole Gosner stage, tadpole size), we assumed a Gaussian distribution and therefore fitted Linear Mixed Models (LMMs). To provide visual insights into the estimated effects and predicted values of each significant variable on frog abundance while holding other factors at their mean values, we used the “ggpredict” function within the “ggeffects” package (Lüdecke et al., 2021).

## 3 Results

We observed a total of 198 tadpoles from three genera: *Crinia* spp., *Limnodynastes* spp. and *Litoria spp*. (Table 1). Among the tadpoles encountered, *Crinia* spp. were most abundant, followed by *Limnodynastes* spp. *Crinia* spp. was the most widely occurring, being present at 15 different dams and observed during 22 surveys. We recorded *Limnodynastes* spp. at 14 dams and in 19 surveys. We found *Litoria spp*. in only two dams during two surveys. The limited number of *Litoria spp*. individuals encountered precluded species‐level analysis but data for this genus were considered in the overall abundance models.

**TABLE 1:** Tadpole counts and the number of dams with tadpoles (in parentheses) for enhanced and control farm dams.

Dam type	Total tadpoles	*Crinia* spp.	*Limnodynastes* spp.	*Litoria* spp.
Enhanced	183 (14)	116 (12)	65 (11)	2 (2)
Control	15 (4)	9 (3)	6 (3)	0
Total	198 (18)	125 (15)	71 (14)	2 (2)

### 3.1 Total Abundance

**FIGURE 1:**
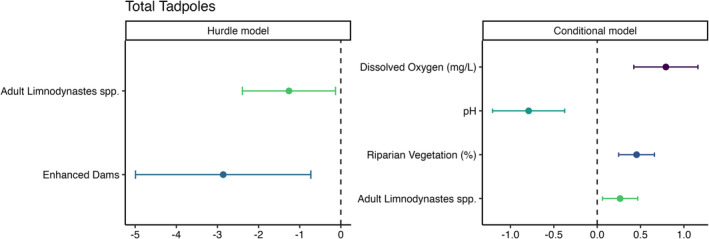
Model effect sizes for tadpole surveys of total tadpole abundance at farm dams. Error bars represent 95% confidence intervals. 95% confidence intervals that cross the zero‐effect line were considered non‐significant. Note, negative values in the hurdle model represent the likelihood of zero and hence, for example, there is a strong positive effect of dam enhancement on the presence of tadpoles.

**FIGURE 2:**
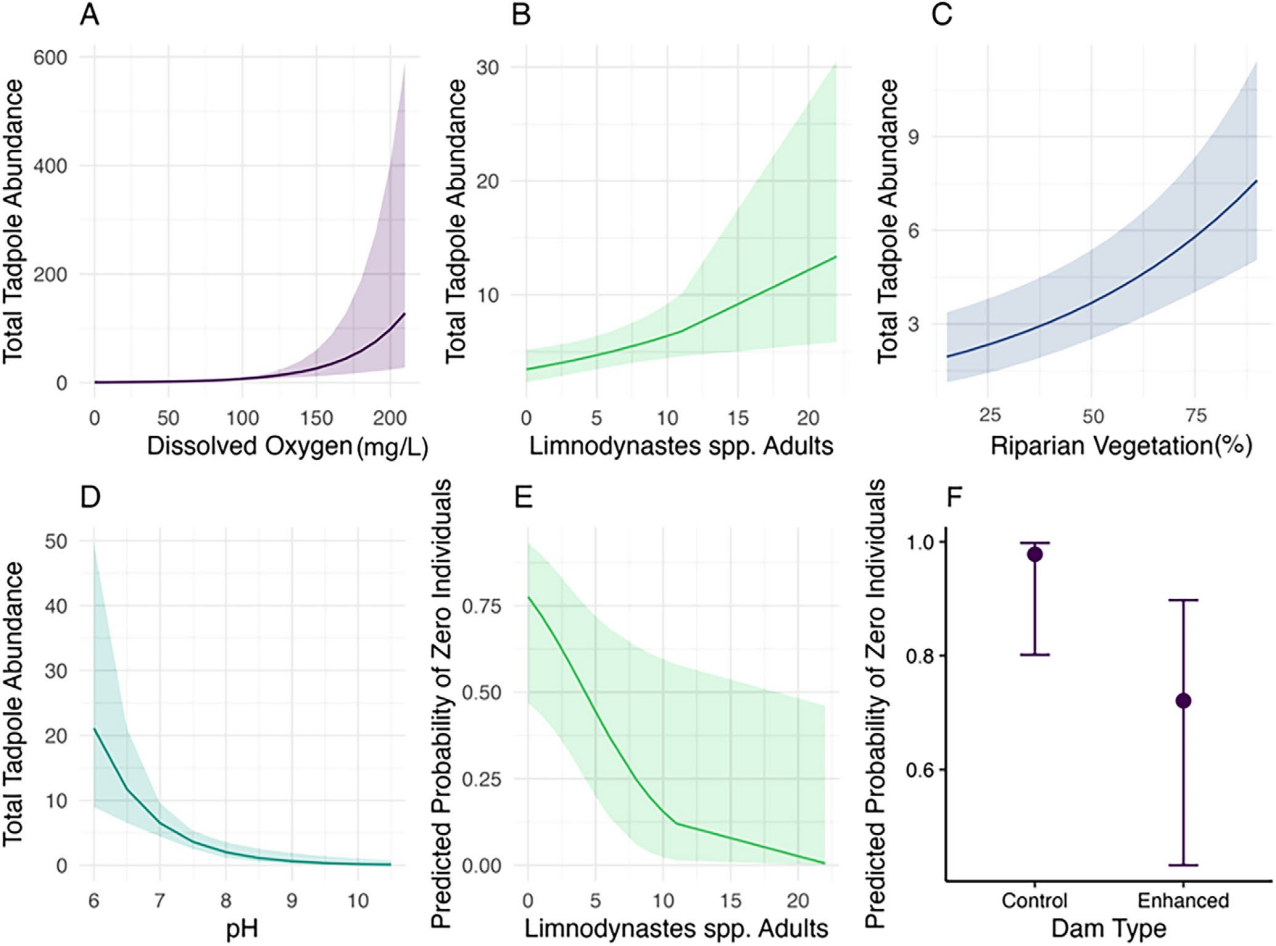
Plots of predicted total tadpole abundance at farm dams in response to (A) Dissolved Oxygen, (B) Adult Limnodynastes spp. presence, (C) Riparian Vegetation, (D) pH. Predictor plots of the likelihood of zero occurrence in response to (E) Adult Limnodynastes spp. Presence and (F) Dam Type. Only those effects considered significant are plotted. Error bands are 95% confidence intervals.

Our analysis revealed several strong relationships between total tadpole abundance and dam variables. Our investigation also revealed that most tadpoles (92%) occurred at enhanced dams (Table 1). In the binary component of the hurdle model, tadpole presence was more likely at enhanced dams and dams with a greater presence of adult *Limnodynastes spp*. (Figures 1 and 2). Our results from the conditional component of the model revealed a positive relationship between tadpole abundance with dissolved oxygen and riparian vegetation cover, and a negative association with pH levels (Figures 1 and 2). The conditional component of the model showed there was a positive relationship between the presence of adult Limnodynastes spp. and total tadpole abundance (Figures 1 and 2).

### 3.2 Individual Species Abundance

**FIGURE 3:**
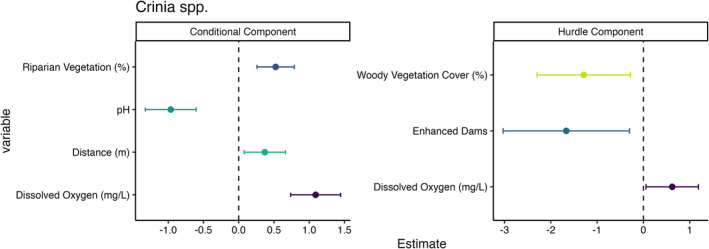
Model effect sizes for tadpole surveys of *Crinia* spp. abundance at farm dams. Error bars represent 95% confidence intervals. 95% confidence intervals that cross the zero‐effect line were considered non‐significant. Note, negative values in the hurdle model represent the likelihood of zero occurrence and hence, for example, there is a strong positive effect of dam enhancement on the presence of *Crinia* spp. tadpoles.

**FIGURE 4:**
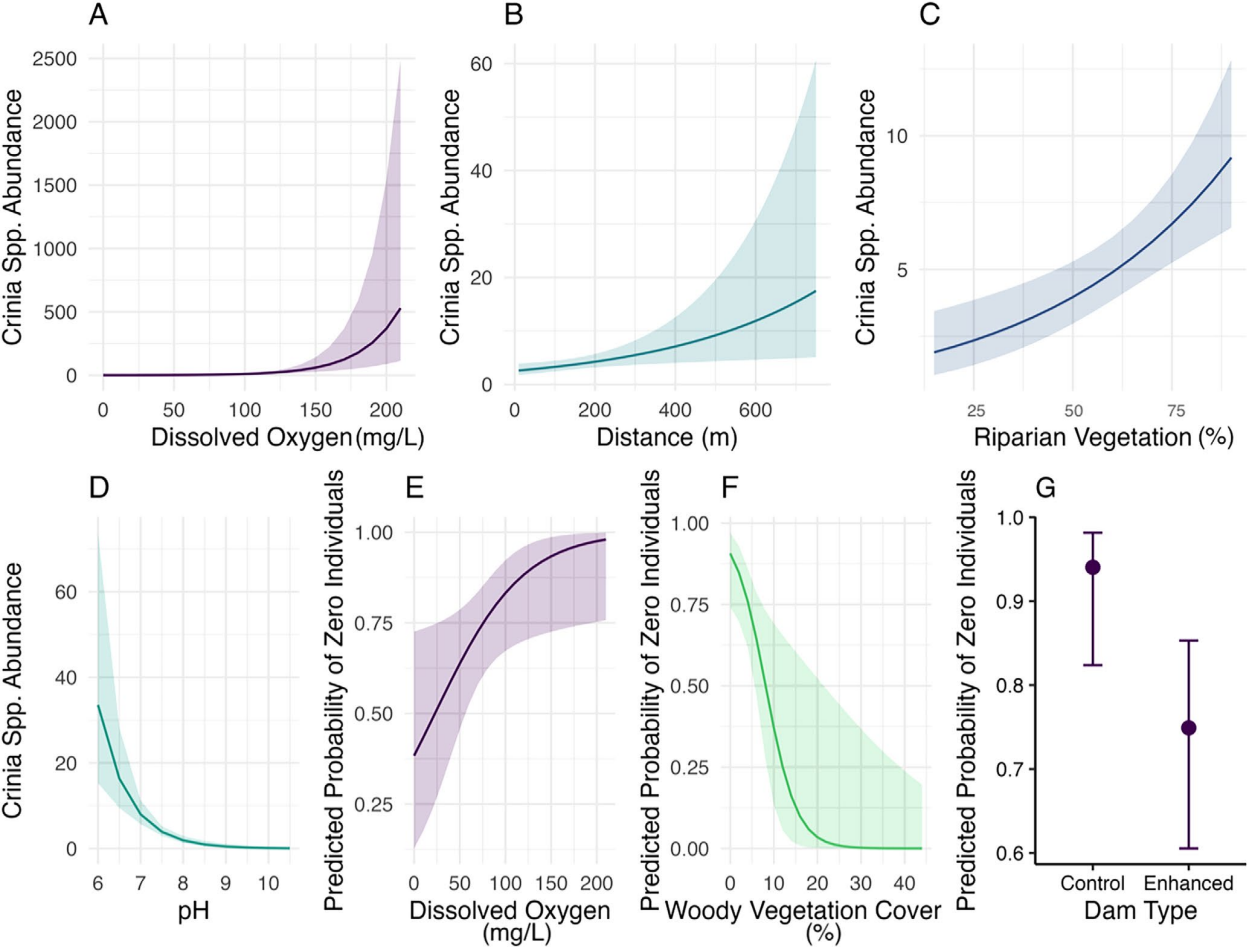
Plots of predicted Crinia spp. tadpole abundance at farm dams in response to (A) Dissolved Oxygen, (B) Distance to the nearest water body (m), (C) Riparian Vegetation, (D) pH. Predictor plots of the likelihood of zero occurrence in response to (E) Dissolved Oxygen, (F) Woody Vegetation Cover and (G) Dam Type. Only those effects considered significant are plotted. Error bands are the standard errors.

**FIGURE 5:**
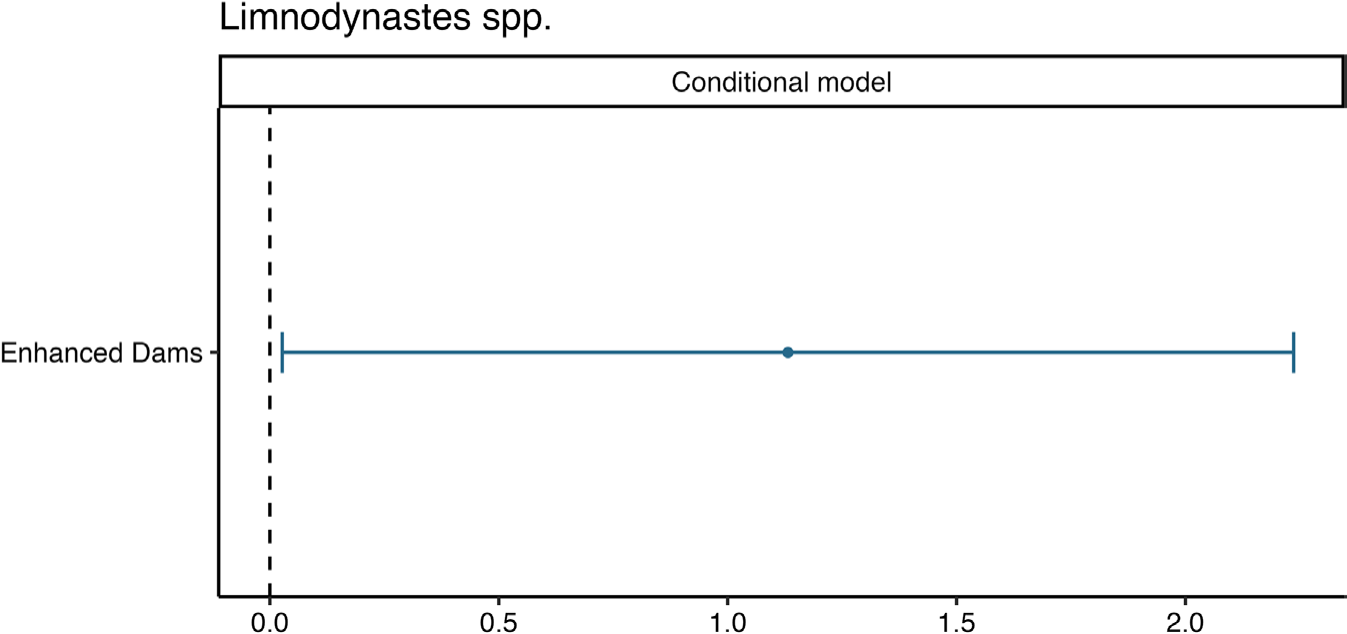
Model effect sizes representing the conditional model for tadpole surveys of Limnodynastes spp. abundance at farm dams. Error bars represent 95% confidence intervals. 95% confidence intervals that cross the zero‐effect line were considered non‐significant. The top ranked model did not include hurdle components.

**FIGURE 6:**
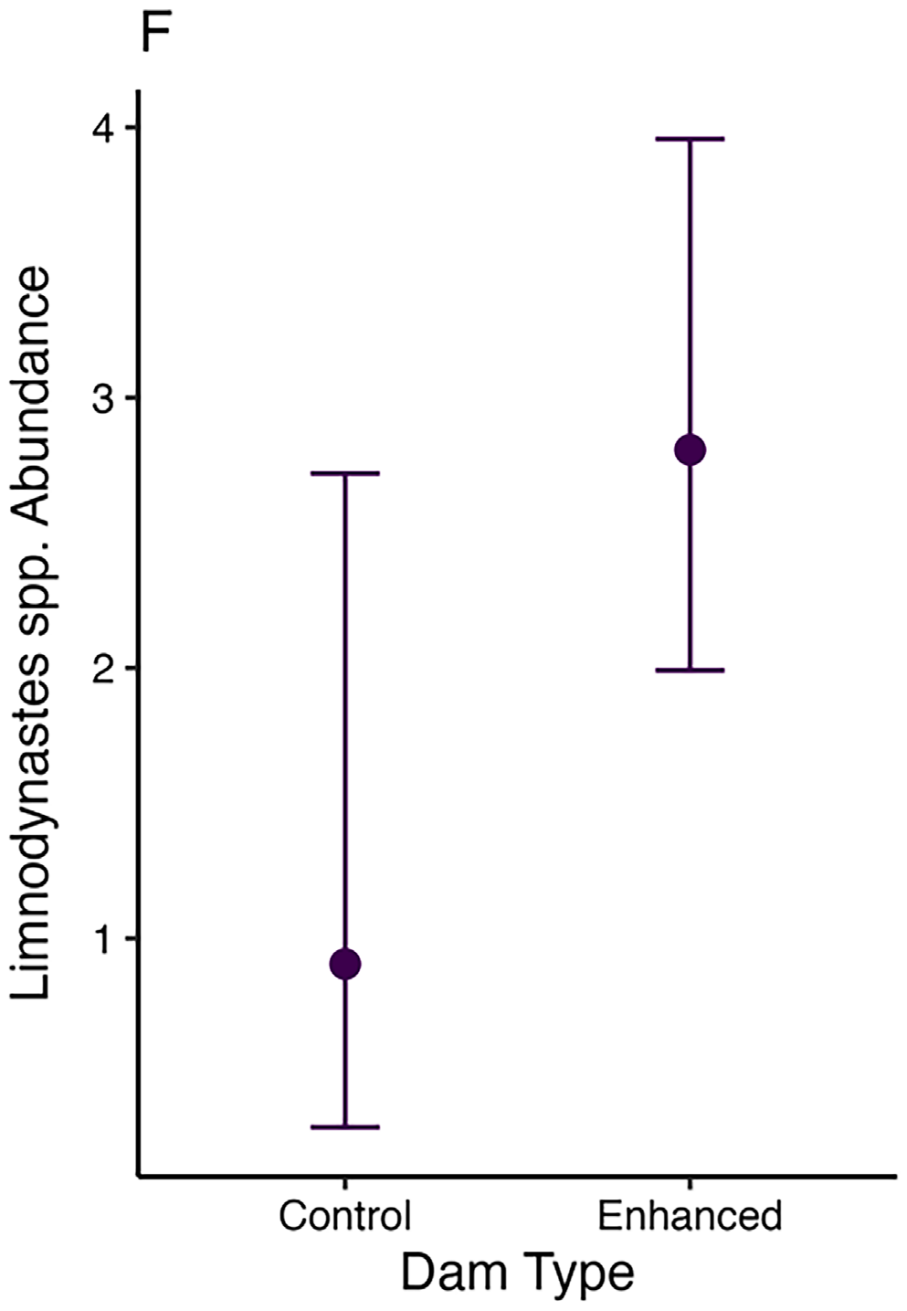
Plots of predicted *Limnodynastes* spp. tadpole abundance at farm dams in response Dam Type. Only those effects considered significant are plotted. Error bands are 95% confidence intervals.

The probability of zero occurrence for *Crinia* spp. increased with higher levels of dissolved oxygen (Figure 3). While enhanced dams and dams with a greater woody vegetation cover within 500 m radius were associated with a decreased the probabilities of zero occurrence (Figure 3). The conditional abundance of *Crinia* spp. showed an increase with rising dissolved oxygen levels, increasing distances to the nearest waterbody and greater riparian vegetation cover (Figure 3 & Figure 4). *Limnodynastes* spp. conditional abundance was higher in enhanced farm dams than control dams (Figures 5 and 6), with no associations in the hurdle component of the model.

## 4 Tadpole Growth

**FIGURE 7:**
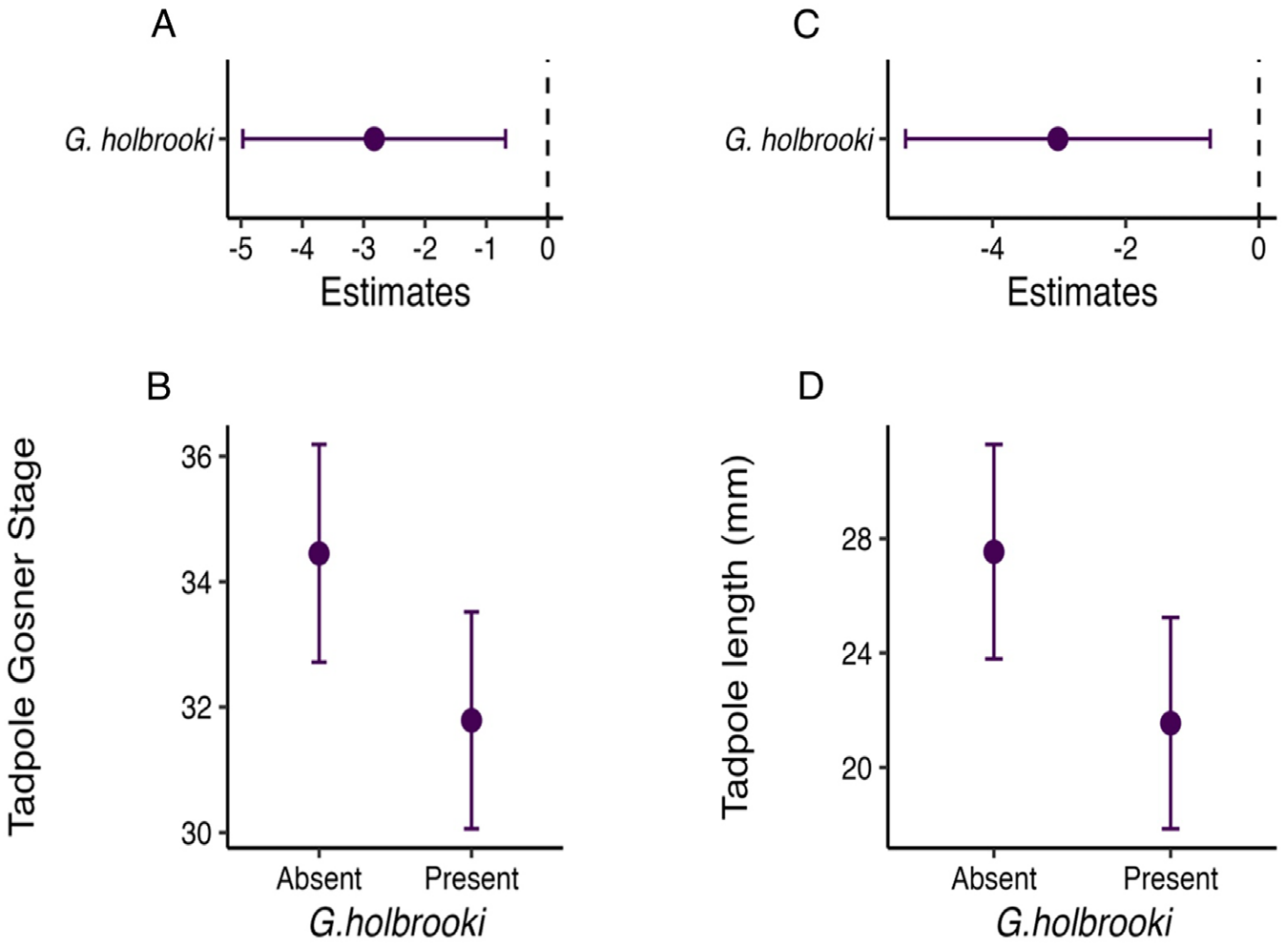
Effect size and predicted plots for tadpole development (A, B) and tadpole length (mm) (C, D) in relation to *G. holbrooki* presence at farm dams. Error bars represent 95% confidence intervals.

*Gambusia holbrooki* presence was negatively associated with tadpole Gosner stage and size (Figure 7). However, there was no discernible influence of *G. holbrooki* presence on tadpole presence or abundance.

## 5 Discussion

Our findings demonstrate the substantial positive effects of enhancing farm dams on the presence of tadpoles. The difference in tadpole abundance between enhanced and control dams suggests that dam management interventions and promoting higher quality habitat can benefit frog populations in agricultural landscapes. Furthermore, our study suggests that dams with better water quality and greater vegetation cover tend to support more abundant tadpole populations. Additionally, we found dams containing *G. holbrooki* , an introduced predator of tadpoles (Reynolds, 2009), typically had smaller, less developed tadpoles. Although no significant differences were detected in tadpole growth rates between the two dam types, implementing habitat enhancements, such as fencing farm dams, may promote tadpole recruitment.

The conversion of native habitats to agricultural land imposes significant pressures on amphibians, and its effects can include habitat fragmentation, agricultural runoff, and land‐use intensification (With et al. 2024; Gillespie et al. 2020). These changes reduce vegetation cover, alter hydrological regimes, and increase exposure to agrochemicals, limiting resources for tadpoles and increasing predation risks (Moreira, de Castilhos, and Castroviejo‐Fisher 2020). Within these modified landscapes, vegetation cover plays an important role in supporting tadpole development. Riparian vegetation was positively associated with total tadpole abundance and *Crinia*. spp. abundance, consistent with its role as a key habitat feature for adult frogs, providing breeding sites, shelter, and food resources that foster population growth and survival (Parris and McCarthy 1999; Rodríguez‐Mendoza and Pineda 2010; Ribeiro, Lima, and Magnusson 2012). Our results also indicate that the chance of tadpoles at dams with no vegetation is zero (Figure 4). However, while the impact of riparian vegetation is clear, the role of aquatic vegetation is more nuanced and may confound sampling efficiency. This is exemplified by the negative association between *Crinia*. spp. presence and aquatic vegetation. This association implies that although aquatic vegetation may not exert a substantial influence on tadpole abundance, it could impose constraints on their presence. These constraints might stem from resource competition or altered environmental conditions influenced by the attributes of aquatic vegetation (Tavares‐Junior, Eskinazi‐Sant'Anna, and Silvério Pires 2020).

We evaluated the role of water quality in shaping the dynamics of tadpole populations within farm dams. We found a relationship between dams with increased pH levels and reduced tadpole abundance. This suggests that, within the context of farm dams, higher pH levels might pose challenges to tadpole development and overall reproductive success (Tavares‐Junior, Eskinazi‐Sant'Anna, and Silvério Pires 2020). In contrast, dissolved oxygen emerged as an important factor influencing tadpole populations in our study, with a positive relationship between dissolved oxygen levels and total tadpole abundance, *Limnodynastes* spp. abundance, as well as *Crinia* spp. abundance. This finding aligns with previous work by Dodd (2009), which suggested that dissolved oxygen not only influences tadpole abundance but also promotes their growth. However, while dissolved oxygen was positively associated with *Crinia spp*. abundance, it was negatively associated with their occurrence, making the interpretation of these results challenging. This discrepancy highlights the need for further research to understand the underlying mechanisms.

Our results contained evidence of a negative impact of the invasive species *G. holbrooki* on tadpole growth within farm dams. While the presence of *G. holbrooki* did not impact tadpole presence or abundance, it had substantial effects on tadpole length and Gosner stage (Gosner 1960). *Gambusia holbrooki* is a known predator of tadpoles and larvae, as well as a potential competitor for resources, potentially making it a limiting factor for tadpole growth and survival (Ruiz‐Navarro et al. 2013; Vannini et al. 2018). Our results align with existing research that demonstrates that tadpole populations exhibit phenotypic plasticity, where environmental factors influence development (Steiner and van Buskirk 2008; Vences et al. 2002). Predator presence, as found in our study, is associated with slower development rates, echoing similar observations in other amphibian studies (Relyea, Schoeppner, and Hoverman, 2005). The implications of *G. holbrooki* on frog populations are concerning, with recent listings designating it as a key threatening process for frogs in NSW and implicating it in the decline of over 15 frog species in Australia (Lintermans, 2007). The removal of *G. holbrooki* from farm dams should be considered given its potential beneficial impact on tadpoles. However, this should be done with care, whether through mechanical means or pesticide application, due to the potentially harmful effects of the removal process on frogs and broader ecological consequences (Ruiz‐Navarro et al. 2013).

Despite the presence of positive relationships between adult abundance and tadpole presence, our study revealed unexpectedly low numbers of tadpoles and reduced species richness compared to adult populations. Littlefair et al. (2024) found approximately 6000 adults across 2 years compared to our 200 tadpoles across 1 year. This disparity suggests significant constraints on tadpole survival and development within farm dam ecosystems Alternatively, it may reflect challenges in effectively sampling or detecting tadpoles, as their apparent rarity seems inconsistent with the high number of adult frogs observed. Our finding prompts consideration of the potential risk of ecological traps in farm dams, where adults may breed in habitats that ultimately fail to support the successful development of their offspring (Battin 2004; Robertson and Hutto, 2006). This may be a particular problem in control dams, where the habitat conditions, characterized by inadequate vegetation cover and poor water quality, may reduce tadpole survival, as observed in most control dams that lacked tadpoles (Dodd Jr., 2009; Tavares‐Junior, Eskinazi‐Sant'Anna, and Silvério Pires 2020), potentially inhibiting successful reproduction despite the presence of breeding adults (Sun et al. 2021). The identification of potential ecological traps within farm dams emphasizes the importance of utilizing a variety of survey techniques and strengthens the argument for surveys across different life stages to fully understand the response of organisms to environmental change and management interventions.

While this study provides important insights into the factors influencing tadpole populations, several caveats must be considered in interpreting our results and planning future research. First, the influence of aquatic and riparian vegetation on sampling efficiency could be further explored. Sanders et al. (2015) noted that the odds of tadpoles being captured were significantly higher in areas of high‐density vegetation, suggesting that vegetation may mask or exaggerate true tadpole densities. Additionally, our methods did not account for mortality rates between sampling periods. Variations in tadpole density could inform assessments of survival rates and habitat suitability, and the potential for higher mortality among smaller tadpoles might skew perceptions of population growth rates. However, the generally higher densities observed in enhanced dams suggest that mortality might be less of an issue in these environments. Future studies should consider these factors to refine our understanding of tadpole population dynamics and the ecological health of aquatic habitats.

### 5.1 Conclusion

Our study sheds light on the potential for farm dams to act as important breeding habitat for amphibians. It suggests that management interventions, such as enhancing farm dams through measures like increasing vegetation cover and improving water quality, may have a positive impact on frog populations by creating more favorable breeding habitats. We found that vegetation cover and water quality influence tadpole abundance, while the presence of *G. holbrooki* negatively impacts tadpole growth. Surprisingly, despite abundant calling adult frogs, tadpole abundance was lower than expected, especially at control dams. Proactive habitat management strategies offer farmers a means to actively contribute to the conservation of farmland frog species, thus promoting their persistence in agricultural landscapes.

## Author Contributions

**Michelle Littlefair:** conceptualization (lead), data curation (lead), formal analysis (equal), funding acquisition (equal), investigation (lead), methodology (lead), project administration (equal), resources (equal), validation (equal), visualization (equal), writing – original draft (lead), writing – review and editing (equal). **Ben C. Scheele:** conceptualization (equal), funding acquisition (equal), methodology (supporting), project administration (equal), resources (equal), supervision (equal), validation (equal), visualization (equal), writing – review and editing (lead). **David Lindenmayer:** conceptualization (equal), funding acquisition (lead), project administration (equal), resources (equal), supervision (equal), writing – review and editing (equal). **Maldwyn J. Evans:** data curation (supporting), formal analysis (lead), methodology (supporting), supervision (equal), validation (lead), writing – review and editing (equal).

## Conflicts of Interest

The authors declare no conflicts of interest.

## Supporting information


Appendix S1.

Appendix S2.

Appendix S3.

## Data Availability

The data are fully available upon request from the corresponding author. Please find the mandatory data for review within the following private link. https://figshare.com/s/45b8c01fdb747ce8a787.
