# Supplementary material for: Enhancing Farm Dams Increases Tadpole Abundance
Source: Ecol Evol. 2025 Jan 19;15(1):e70803. doi: 10.1002/ece3.70803 (PMC11742428; doi:10.1002/ece3.70803)
Supplement: Supplementary file 1 — Appendix S1. [file ECE3-15-e70803-s002.docx]

| **Category** | **Variable** | **Description** | **Control Dam Mean ± SD** | **Enhanced Dam  Mean ± SD** |
| --- | --- | --- | --- | --- |
| Dam Type | Enhanced | N = 28. Management implemented within the last 3-20 years. Options include full livestock exclusion through fencing or partial fencing allowing restricted access. Native shrubs and trees planted between fence and dam. | na | na |
|  | Control | N = 24. No management intervention. Follows neighbouring paddocks' practices, including dryland cropping, cattle or sheep grazing, or both. | na | na |
| Water Quality | pH | The pH of the dam’s littoral zone, measured in three quadrats, two metres from edge. | 7.49 ± 0.55 | 7.46 ± 0.76 |
|  | Dissolved Oxygen | The dissolved oxygen of the dam’s littoral zone, measured in three quadrats, two metres from edge. | 72.45 ± 21.75 | 77.79 ± 35.33 |
|  | Total Dissolved Solids | The total dissolved solids of pond littoral zone, measured in three quadrats, two metres from edge. | 153.43 ± 99.77 | 126.96± 69.52 |
| Dam Vegetation | Aquatic Vegetation Cover | The average percent cover of total aquatic vegetation, including attached floating, aquatic vegetation extending over 1m, aquatic vegetation reaching under 1m, and horizontal algae cover on the surface of the dam. | 23.25± 34.73 | 26.32 ± 35.62 |
|  | Riparian Vegetation Cover | The average percent cover of total riparian vegetation, including ground vegetation, woody vegetation, rushes, sedges and forbs. | 45.5 ± 34.64 | 51.57 ± 40.45 |
| Landscape | Distance to nearest waterbody (m) | The distance in metres to the nearest mapped waterbody. Calculated using the distance tool in ArcGIS | 210.29 ± 183.75 | 145.54 ± 87.80 |
|  | Topographic Wetness Index | The wetness of the 500m radius around each site, calculated as ln(*α*/tan *β*), where *α* is the size of UCA per unit length of contour (i.e., pixel resolution of 10 m) and *β* is the slope gradient of the cell. | 8.77 ± 0.66 | 10.21 ± 11.15 |
|  | Woody Vegetation Cover 2019 | The Woody Vegetation Cover (WCF), within 500m radius around each site. The data queries are derived annual mapping of woody vegetation cover across Australia for 2000-2018 by Liao et al (2020) based on annual geomedian Landsat reflectance data contained in DEA. Thus, these data are inherently of annual resolution. An online data explorer is available via http://anuwald.science/tree | 3.38 ± 1.91 | 4.93 ± 5.94 |
| Fauna | *Crinia spp.* | The abundance of calling and visually observed *Crinia spp.* adults. | 0.21 ± 0.41 | 9.61 ± 14.21 |
|  | *Limnodynastes spp.* | The abundance of calling and visually observed *Limnodynastes spp.* adults. | 2.25 ± 3.82 | 3.18 ± 4.65 |
|  | *Gambusia holbrooki* | The presence of *Gambusia holbrooki* in the farm dams recorded as a binary (0,1). | 0.21 ± 0.41 | 0.50 ± 0.51 |

Appendix 1.  Habitat variables used in tadpole abundance analyses including the mean and standard deviation for each variable at control and enhanced dams.
