# Supplementary figures and images for: Enhancing Farm Dams Increases Tadpole Abundance
Source: Ecol Evol. 2025 Jan 19;15(1):e70803. doi: 10.1002/ece3.70803 (PMC11742428; doi:10.1002/ece3.70803)

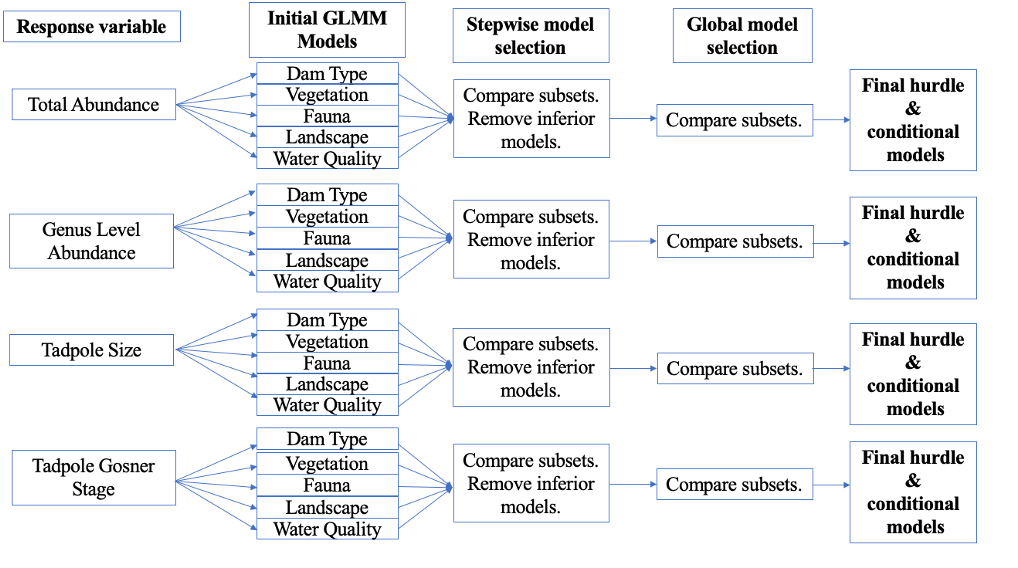

Supplement: Supplementary file 2 — Appendix S2. [file ECE3-15-e70803-s001.png]
