# Supplementary material for: Enhancing Farm Dams Increases Tadpole Abundance
Source: Ecol Evol. 2025 Jan 19;15(1):e70803. doi: 10.1002/ece3.70803 (PMC11742428; doi:10.1002/ece3.70803)
Supplement: Supplementary file 3 — Appendix S3. [file ECE3-15-e70803-s003.docx]

Appendix 3. Model Selection and Results Summary for Tadpole Abundance and Species-Specific Analyses

Total tadpole abundance. Competing models within delta AICc < 2. We chose the simplest model to interpret our effects (bolded).

| cond (MgVeg) | cond (DO) | cond (L.tas) | cond (pH) | cond(TWI) | zi (AqVeg) | zi (C.par) | zi(DO) | zi (L.tas) | zi (Type) | df | logLik | AICc | Δ | Pseudo R^2^ |
| --- | --- | --- | --- | --- | --- | --- | --- | --- | --- | --- | --- | --- | --- | --- |
| 0.45 | 0.79 | 0.26 | -0.79 | -0.29 |  | 1.30 |  | -1.26 | + | 12 | -114.72 | 256.88 | 0.00 | 0.33 |
| 0.45 | 0.79 | 0.26 | -0.79 | -0.28 | -0.57 | 1.43 |  | -1.36 | + | 13 | -113.53 | 257.10 | 0.23 | 0.33 |
| **0.48** | **0.80** | **0.29** | **-0.79** |  |  | **1.30** |  | **-1.26** | **+** | **11** | **-116.27** | **257.40** | **0.53** | **0.32** |
| 0.48 | 0.80 | 0.29 | -0.79 |  | -0.57 | 1.43 |  | -1.36 | + | 12 | -115.07 | 257.58 | 0.70 | 0.32 |
| 0.45 | 0.79 | 0.26 | -0.79 | -0.28 |  | 1.30 | 0.39 | -1.18 | + | 13 | -114.04 | 258.12 | 1.24 | 0.33 |
| 0.48 | 0.80 | 0.29 | -0.79 |  |  | 1.30 | 0.39 | -1.18 | + | 12 | -115.58 | 258.59 | 1.72 | 0.32 |

L. tasmaniensis tadpole abundance. Competing models within delta AICc < 2. We chose the simplest model to interpret our effects (bolded).

| cond (Dist) | cond (TWI) | Cond (Type) | zi  (Dist) | zi (DO) | zi (Type) | df | logLik | AICc | Δ | Pseudo R^2^ |
| --- | --- | --- | --- | --- | --- | --- | --- | --- | --- | --- |
| -0.44 | -0.22 | + |  |  |  | 7 | -81.29 | 177.74 | 0.00 | 0.07 |
| -0.46 |  | + |  |  |  | 6 | -82.56 | 177.99 | 0.25 | 0.05 |
|  |  | **+** |  |  |  | **5** | **-84.11** | **178.81** | **1.07** | **0.04** |
| -0.44 | -0.22 | + |  |  | + | 8 | -80.73 | 178.97 | 1.23 | 0.07 |
| -0.46 |  | + |  |  | + | 7 | -82.00 | 179.18 | 1.44 | 0.05 |
|  | -0.22 | + |  |  |  | 6 | -83.16 | 179.19 | 1.45 | 0.05 |
| -0.44 | -0.22 | + |  | -0.47 |  | 8 | -81.02 | 179.56 | 1.82 | 0.07 |
| -0.44 | -0.22 | + | 0.69 |  |  | 8 | -81.08 | 179.69 | 1.94 | 0.07 |

C. parinsignifera tadpole abundance. Competing models within delta AICc < 2. We chose the simplest model to interpret our effects (bolded).

| cond (MgVeg) | cond (Dist) | cond (DO) | cond (pH) | cond (WVC) | cond (Type) | zi (AqVeg) | zi (MgVeg) | zi (DO) | zi (TDS) | zi (WVC) | zi (Type) | df | logLik | AICc | Δ | Pseudo R^2^ |
| --- | --- | --- | --- | --- | --- | --- | --- | --- | --- | --- | --- | --- | --- | --- | --- | --- |
| **0.52** | **0.37** | **1.09** | **-0.964** |  |  |  |  | **0.62** |  | **-1.29** | **+** | **9** | **-90.78** | **201.47** | **0.00** | **0.35** |
| 0.52 | 0.37 | 1.09 | -0.96 |  |  |  | -0.37 | 0.61 |  | -1.22 | + | 10 | -89.98 | 202.33 | 0.85 | 0.36 |
| 0.52 | 0.37 | 1.09 | -0.96 |  |  | -0.34 |  | 0.59 |  | -1.34 | + | 10 | -90.00 | 202.37 | 0.89 | 0.36 |
| 0.52 | 0.37 | 1.09 | -0.96 |  |  |  |  | 0.67 | 0.41 | -1.17 | + | 10 | -90.28 | 202.93 | 1.46 | 0.36 |
| 0.52 | 0.37 | 1.09 | -0.96 |  |  | -0.41 |  | 0.66 | 0.54 | -1.21 | + | 11 | -89.20 | 203.27 | 1.80 | 0.36 |
| 0.53 | 0.36 | 1.03 | -0.91 | 0.06 |  |  |  | 0.62 |  | -1.29 | + | 10 | -90.48 | 203.32 | 1.84 | 0.36 |
| 0.50 | 0.40 | 1.05 | -0.93 |  | + |  |  | 0.62 |  | -1.29 | + | 10 | -90.52 | 203.42 | 1.94 | 0.36 |
